# Glucagon-like Peptide-1 Receptor Analogues for the Treatment of Obesity

**DOI:** 10.17925/EE.2022.18.1.43

**Published:** 2022-06-18

**Authors:** David M Williams, Matthew Staff, Stephen C Bain, Thinzar Min

**Affiliations:** 1. Department of Diabetes and Endocrinology, Singleton Hospital, Swansea Bay University Health Board, Swansea, UK; 2. Diabetes Research Group, Swansea University Medical School, Swansea University, Swansea, UK; 3. Department of Diabetes and Endocrinology, Neath Port Talbot Hospital, Swansea Bay University Health Board, Swansea, UK

**Keywords:** Glucagon-like peptide-1 (GLP-1) receptor analogue, liraglutide, semaglutide, obesity, overweight, weight loss, weight management, diabetes mellitus, type 2 diabetes

## Abstract

There is an increasing prevalence of obesity worldwide, associated with significant morbidity and mortality, which frequently reduces quality of life and life expectancy. Consequently, there is a substantial and growing personal and economic burden necessitating the development of more effective therapies for obesity. Glucagon-like peptide-1 receptor analogues (GLP-1RAs) are licensed for the treatment of type 2 diabetes (T2D), and there is substantial evidence that these drugs not only improve cardiovascular outcomes but also promote weight loss. More recent evidence supports the use of the GLP-1RAs liraglutide and semaglutide in people with obesity without T2D. This article discusses the results of the major cardiovascular outcome trials for GLP-1RAs in people with T2D, the SCALE Obesity and Prediabetes study (Effect of liraglutide on body weight in non-diabetic obese subjects or overweight subjects with co-morbidities: SCALE™ - Obesity and Pre-diabetes; ClinicalTrials.gov identifier: NCT01272219; investigating liraglutide) and the STEP studies (Semaglutide treatment effect in people with obesity; assorted studies; investigating subcutaneous semaglutide). We also highlight the importance of a cost-effective approach to obesity pharmacotherapy. Clinicians should consider the use of GLP-1RAs in people with obesity, especially those with T2D or other obesity-related diseases, such as hypertension and dyslipidaemia. Ongoing trials, as well as clinical and cost-effectiveness appraisals, are anticipated over the next 12 months, and their findings may change the current landscape of obesity pharmacotherapy.

Obesity is a growing problem worldwide with extensive health and economic consequences, rendering interventions that encourage weight loss of great interest. Obesity is usually defined as a body mass index (BMI) of 30 kg/m^2^ or above, or 27.5 kg/m^2^ or above in Asian populations. Based on the health survey for England 2019, 28% of adults in England are obese.^1^ This is not unique to the UK, as the prevalence of obesity has increased in every country worldwide over the past 50 years, driven mainly by changes in the global food system and more sedentary lifestyles.^2,3^ From 1999–2000 to 2017–2018, the prevalence of obesity in the USA increased from 30.5% to 42.4%.^4^ In 2016, the World Health Organization (WHO) found that 1.9 billion adults aged over 18 were overweight, which equates to 39% of the population.^5^ Of these adults, 650 million were obese, which equates to 13% of the population.^5^ The disparity between the UK, USA and WHO obesity prevalence could be explained by the low prevalence of obesity in low-income countries.^6^ Despite obesity being preventable and the known economic and health implications, obesity will also be a problem for future generations, as 38 million children under the age of 5 were overweight or obese in 2019.^5^ Treatment, as well as prevention, will therefore continue to be an important intervention.

A raised BMI is associated with an increase in all-cause mortality, largely driven by excess cardiovascular disease.^7^ Obesity is also associated with significant morbidity and mortality from a wide range of health conditions, including various cancers, cerebrovascular disease, hypertension, type 2 diabetes mellitus (T2D), chronic kidney disease, osteoarthritis and back pain. As a result, obesity is associated with long-term physical impairment and reduced quality of life for many people around the world, including those of working age.^8^ In addition, obesity has been identified as an independent risk factor for COVID-19 mortality in hospitalized patients.^9^ The economic impact of obesity is substantial, and the amount spent on the treatment of obesity and T2D in the UK is more than the combined cost of the police, fire service and judicial system.^10^ In 2014/15, obesity-related illness cost the UK National Health Service £6.1 billion.^10^ The estimated medical cost of obesity in the USA ranged from US$147 billion to nearly US$210 billion per year.^4^ The medical cost for people with obesity was US$1,429 higher than medical costs for people with a healthy weight.^4^ Therefore, weight loss interventions have the potential to significantly improve the cost-effectiveness of health services.

Given the health and economic impact of obesity, effective treatment options are of great importance and include lifestyle, pharmacological and surgical interventions. Whilst lifestyle modifications are effective at achieving weight loss, adherence to a healthy lifestyle to maintain weight loss is a challenge.^11^ Surgical intervention is the most effective treatment for long-term weight loss, but is not widely available and is highly invasive, with associated surgical risk and complications.^12^ For this reason, bariatric surgery is reserved for patients for whom lifestyle or pharmacotherapy interventions have failed. Pharmacotherapy is a non-invasive and easily administered intervention that supports weight loss alongside lifestyle modification and can reduce obesity-related health problems and improve quality of life.^13^

## Current drug therapies for obesity

The current European guidelines advocate drug therapy for individuals with a BMI of >30 kg/m^2^ or a BMI >27 kg/m^2^ and with an obesity-related disease such as T2D, hypertension or dyslipidaemia.^13^ Drug therapies currently approved by the European Medicines Agency (EMA) include orlistat, bupropion/naltrexone and liraglutide, which have been observed to support meaningful weight loss.^13^ Meaningful weight loss is generally defined as loss of >5% of total body weight, based on the Look AHEAD (Action for health in diabetes; ClinicalTrials.gov Identifier: NCT00017953) trial, which found that >5% weight loss was associated with improvement in various surrogate markers for cardiovascular disease, including blood pressure and lipids.^11^

Generally, drug therapies for obesity act to induce satiety, reduce hunger and/or reduce fat absorption or catabolism. Orlistat reduces digestion and absorption of fat in the intestine by inhibiting pancreatic lipase, resulting in a weight loss of 2.9–3.4 kg per year.^13^ However, orlistat commonly causes intolerable gastrointestinal side effects, such as steatorrhea and malabsorption of fat-soluble vitamins, which limit its use.^14^ The estimated cost per quality-adjusted life year (QALY) is up to £45,881, limiting its use in the UK following the recommendation by the National Institute for Health and Care Excellence (NICE).^15^

Bupropion/naltrexone is a combination of a dopamine and norepinephrine reuptake inhibitor (bupropion) and an opioid receptor antagonist (naltrexone). These both work centrally to increase satiety and were originally used for nicotine, alcohol and opiate addictions. Bupropion/naltrexone produces weight loss of a mean of 4.4 kg, and side effects include nausea, headaches and dizziness.^16^ However, their use in the UK is not recommended by NICE given the additional cost per QALY of £23,750, which is greater than the usual cost-effectiveness thresholds for such interventions in the UK.^17^

Amongst glucagon-like peptide-1 (GLP-1) receptor analogues (GLP-1RAs), liraglutide 3 mg (Saxenda^®^; Novo Nordisk Inc., Plainsboro, NJ, USA) is recommended by NICE, and semaglutide 2.4 mg (Wegovy^®^; Novo Nordisk Inc., Plainsboro, NJ, USA) has recently received approval from the US Food and Drug Administration (FDA) in the USA for chronic weight management.^18,19^ GLP-1RAs aid weight loss by suppressing appetite to reduce calorie intake, as detailed below. They are generally well tolerated, but common side effects include nausea, vomiting and diarrhoea.^13^ GLP-1RA therapies approved for the management of T2D are an attractive option for the treatment of obesity. These drugs are already used commonly in clinical practice for T2D, and therefore data on their pharmacology, side effects, patient compliance/satisfaction and interactions are already available. Within cardiovascular outcome trials (CVOTs) and dedicated weight loss trials, GLP-1RAs have already shown a significant effect on weight loss and other metabolic outcomes related to obesity, including major adverse cardiovascular events and renal outcomes.^20,21^ In this article, we present the evidence supporting the use of GLP-1RAs in the treatment of obesity in people with or without T2D and discuss ongoing clinical trials of drugs within this class.

## Diabetes and obesity: The role of incretin hormones

The incretin effect was first identified by Nauck et al. 35 years ago.^22^ The incretin effect describes augmented insulin secretion from the pancreas following an oral intake containing carbohydrate. A blunted incretin response was observed in people with T2D.^23^ Subsequently, GLP-1 was recognized as a major player in the incretin system, raising the possibility of manipulating this system to treat people with T2D.^24^ Enteroendocrine L-cells of the small intestine secrete incretin hormones, including GLP-1 and glucose-dependent insulinotropic peptide (GIP), in response to oral glucose or fat. Then, the incretin hormones stimulate the beta cells of the pancreas to secrete insulin and are subsequently degraded by the enzyme dipeptidyl peptidase-4 (DPP-4). Over the past 30 years, this has formed the basis for the development and clinical use of two major drug classes in the treatment of T2D: GLP-1RAs and DPP-4 inhibitors.^25^ The use of GLP-1RAs in people with T2D has become commonplace over the past 15 years, with multiple drugs in this class commercially available, including exenatide, liraglutide, lixisenatide, dulaglutide and semaglutide. Their use is associated with multiple metabolic benefits in people with T2D, including improved glycaemic control, blood pressure, lipid profiles, body weight and, notably, major adverse cardiovascular events.^21^ More recently, dual GIP and GLP-1 analogues have been developed and investigations are currently under way into the treatment of people with T2D and obesity.^26^

The broad health benefits seen in people with T2D are a result of the widespread expression of the GLP-1 receptor around the body. The GLP-1 receptor has been identified in the pancreatic islets, gastrointestinal tract, kidney, lung, heart, and centrally within the hypothalamus and pituitary. The influence of GLP-1RAs on weight is secondary to the combined impact of chemically mediated satiety within the hypothalamus and mechanical satiety by reducing gastric emptying to engender satiation.^27^ Whilst some authors speculate that GLP-1RAs increase energy expenditure through brown fat activation and reduced peripheral lipid storage, this has not been demonstrated in human subjects.^28,29^ The commonest side effects associated with GLP-1RAs include nausea, vomiting and gastrointestinal disturbance, which reduce food intake and support weight loss.^30^

## Reviewing the evidence: GLP-1RAs in clinical trials

As clinical trials of people with T2D have demonstrated the improved glycaemic control and body weight loss associated with GLP-1RAs, there is major interest in the use of these drugs for weight loss in people with obesity. To date, dedicated CVOTs have been undertaken for exenatide (Exenatide study of cardiovascular event lowering trial [EXSCEL]: A trial to evaluate cardiovascular outcomes after treatment with exenatide once weekly in patients with type 2 diabetes mellitus; ClinicalTrials.gov identifier: NCT01144338),^31^ lixisenatide (A randomized, double-blind, placebo-controlled, parallel-group, multicenter study to evaluate cardiovascular outcomes during treatment with lixisenatide in type 2 diabetic patients after an acute coronary syndrome [ELIXA]; ClinicalTrials.gov identifier: NCT01147250),^32^ liraglutide (A long-term, multi-centre, international, randomised double-blind, placebo-controlled trial to determine liraglutide effects on cardiovascular events [LEADER]; ClinicalTrials.gov identifier: NCT01179048),^33^ dulaglutide (Researching cardiovascular events with a weekly incretin in diabetes [REWIND]; ClinicalTrials.gov: NCT01394952)^34^ and semaglutide (A long-term, randomised, double-blind, placebo-controlled, multinational, multi-centre trial to evaluate cardiovascular and other long-term outcomes with semaglutide in subjects with type 2 diabetes [SUSTAIN™ 6 - Long-term Outcomes]; ClinicalTrials.gov identifier: NCT01720446.

**Table 1:** Summary of glucagon-like peptide-1 receptor analogues in cardiovascular outcome trials

Drug	Trial	Population	Baseline body weight/BMI	Absolute weight loss/BMI change*	Weight loss/BMI change versus placebo
Exenatide (2 mg SC weekly)	EXSCEL^31^ (NCT01144338) [n=14,752]. Median follow-up: 3.2 years	HbA1c: 8.0% (7.3, 8.9) (64 ± 9 mmol/mol). Age: 61.9 ± 9.4 years	BMI: 31.8 kg/m^2^ (28.2, 36.2)	–	Weight loss: -1.3 kg
Lixisenatide (20 μg SC daily)	ELIXA^32^ (NCT01147250) [n=6,068]. Median follow-up: 25 months	HbA1c: 7.6 ± 1.3% (60 ± 14 mmol/mol). Age: 60.5 ± 9.6 years	Body weight: 85.1 ± 19.6 kg BMI 30.2 ± 5.8 kg/m^2^	Weight loss (25 months): -0.6 kg	Weight loss: -0.6 kg
Liraglutide (1.8 mg SC daily)	LEADER^33^ (NCT01179048) [n=9,340]. Median follow-up: 3.8 years	HbA1c: 8.7 ± 1.6% (72 ± 17 mmol/mol). Age: 64.2 ± 7.2 years	Body weight: 91.9 ± 21.2 kg BMI: 32.5 ± 6.3 kg/m^2^	–	Weight loss: -2.3 kg
Dulaglutide (1.5 mg SC weekly)	REWIND^34^ (NCT01394952) [n=9,901]. Median follow-up: 5.4 years	HbA1c: 7.3 ± 1.1% (56 ± 12 mmol/mol). Age: 66.2 ± 6.5 years	BMI: 32.3 ± 5.7 kg/m^2^	Weight loss (5.4 years): -3.0 kg BMI (5.4 years): -1.1 kg/m^2^	Weight loss: -1.5 kg BMI (5.4 years): -0.5 kg/m^2^
Semaglutide (0.5–1.0 mg SC weekly)	SUSTAIN-6^35^ (NCT01720446) [n=3,297]. Median follow-up: 2.1 years	HbA1c: 8.7 ± 1.5% (72 ± 16 mmol/mol). Age: 64.6 ± 7.4 years	Body weight: 91.2 ± 20.6 kg BMI: 32.8 ± 6.2 kg/m^2^	Weight loss (2 years): 0.5 mg: -3.6 kg 1.0 mg: -4.9 kg	Weight loss: 0.5 mg: -2.9 kg 1.0 mg: -4.3 kg
Semaglutide (14 mg PO daily)	PIONEER-6^36^ [n=3,183]. Median follow-up: 15.9 months	HbA1c: 8.2 ± 1.6% (66 ± 17 mmol/mol) Age: 66 ± 7 years	Body weight: 91.0 ± 21.4 kg BMI: 32.3 ± 6.6 kg/m^2^	Weight loss (15.9 months): -4.2 kg	Weight loss: -3.4 kg

*Data represent the median (Q1, Q3) or the mean ± standard deviation of the baseline HbA1c, age, body weight or BMI.*

*Data about absolute weight loss/BMI change was missing for exenatide and lixisenatide.

*BMI = body mass index; HbA1c = glycated haemoglobin; PO = by mouth; SC = subcutaneous.*

A trial investigating the cardiovascular safety of oral semaglutide in subjects with type 2 diabetes [PIONEER-6]; ClinicalTrials.gov identifier: NCT02692716).^35,36^ All of these CVOTs included only participants with T2D and with varying degrees of cardiovascular risk. As shown in *Table 1*, the average baseline BMI of participants in these trials was broadly similar (30.2–32.8 kg/m^2^), though there was some variability in baseline glycated haemoglobin (HbA1c; 7.3–8.7%, 56–72 mmol/mol). Whilst all CVOTs investigating the safety of GLP-1RAs showed a consistent reduction in body weight versus placebo, the degree of excess weight loss associated with exenatide (1.3 kg) or lixisenatide (0.6 kg) was relatively small compared with liraglutide (2.3 kg) or semaglutide (2.9–4.3 kg). Moreover, differences in follow-up duration limit comparison between studies (15.9 months – 5.4 years). This may be important, as the temporal effect of weight loss associated with each of these GLP-1RAs is different, though generally seen in the early phase of treatment. Nevertheless, as these trials were not designed primarily to evaluate weight loss, the results should be interpreted with caution.

The SURPASS programme investigating tirzepatide, a once-weekly dual GIP/GLP-1 agonist, has demonstrated promising results for both HbA1c reduction and weight reduction.^37,38^ The SURPASS-1 (A study of tirzepatide [LY3298176] in participants with type 2 diabetes not controlled with diet and exercise alone; ClinicalTrials.gov: NCT03954834) clinical trial observed the use of tirzepatide in people with T2D, which resulted in a dose-dependent reduction in body weight.^37^ After 40 weeks, participants using tirzepatide 5 mg, 10 mg and 15 mg had a placebo-adjusted weight reduction of 6.3 kg, 7.1 kg and 8.8 kg, respectively. Participants using the highest 15-mg dose had a mean total body weight reduction of 11.0%. There was also a significant reduction in mean HbA1c of 2.11% (23 mmol/mol) over the trial period. The SURPASS-2 clinical trial investigating tirzepatide against semaglutide (A study of tirzepatide [LY3298176] versus semaglutide once weekly as add-on therapy to metformin in participants with type 2 diabetes; ClinicalTrials.gov: NCT03987919) demonstrated that tirzepatide at all doses was non-inferior and superior to semaglutide in both HbA1c and weight reduction.^38^ At 40 weeks, mean weight loss in tirzepatide groups (5 mg, 10 mg and 15 mg) were -7.6 kg, -9.3 kg and -11.2 kg, respectively, compared with -5.7 kg with semaglutide. A total of 15–35% of participants receiving tirzepatide achieved 15% weight loss, compared with 8% of those receiving semaglutide.

## Obesity without diabetes: A role for GLP-1RAs?

There is evidence that the incretin response is blunted in people with obesity in the absence of underlying T2D, implying therapeutic potential in this often difficult-to-treat group.^39^ As weight loss outcomes in trials involving people with T2D are consistently promising, further clinical trials have investigated some drugs in this class to support weight loss in people without T2D.

The use of liraglutide 3.0 mg daily was explored in the SCALE Obesity and Prediabetes study (Effect of liraglutide on body weight in non-diabetic obese subjects or overweight subjects with co-morbidities: SCALE™ - Obesity and Pre-diabetes; ClinicalTrials.gov identifier: NCT01272219) over 56 weeks in 3,731 participants with a BMI ≥30 kg/m2 or ≥27 kg/m^2^ and an obesity-related disease without T2D.^40^ Participants had a baseline mean body weight of 106.2 ± 21.2 kg and BMI of 38.3 ± 6.4 kg/m^2^. The study observed a placebo-adjusted weight loss of 5.6 kg and a BMI reduction of 2.0 kg/m^2^. The mean additional body weight reduction in those receiving liraglutide was 5.4%, with 63.2% (versus 27.1%) and 33.1% (versus 10.6%) of participants attaining at least 5% or 10% body weight reduction, respectively, compared with the placebo group. Subsequently, Saxenda^®^ (liraglutide) has been approved for weight loss by the EMA, FDA and NICE.^41–43^

**Table 2:** Summary of the STEP programme (published studies)^45–48^

Trial^Ref^	Population	Comparator to semaglutide 2.4 mg	Results			
			Mean weight changes from baseline at 68 weeks	Proportion of participants achieving (semaglutide versus placebo)		
				>5% weight loss	>10% weight loss	>15% weight loss
STEP-1^45^ (n=1,961)	Age: 46.5 ± 12.5 years Weight: 105.3 ± 22.1 kg BMI: 37.9 ± 6.6 kg/m^2^ Prediabetes: 43.7%	Placebo	-14.9% versus -2.4%	86.4% versus 31.5%	69.1% versus 12.0%	50.5% versus 4.9%
STEP-2^46^ (n=1,210)	Age: 55.2 ± 11 years Weight: 99.8 ± 21.5 kg BMI: 35.7 ± 6.3 kg/m^2^ Duration of T2D: 8.0 ± 6.1 years	Semaglutide 1 mg, placebo	-9.6% versus -7.0% (semaglutide 1 mg) versus -3.4% (placebo)	68.8% versus 57.1% (semaglutide 1 mg) versus 28.5% (placebo)	45.6% versus 28.7% (semaglutide 1 mg) versus 8.2% (placebo)	25.8% versus 13.7% (semaglutide 1 mg) versus 3.2% (placebo)
STEP-3^47^ (n=611)	Age: 46 ± 13 years Weight: 105.8 ± 22.9 kg BMI: 38.0 ± 6.7 kg/m^2^	Placebo	-16.0% versus -5.7%	86.6% versus 47.6%	75.3% versus 27.0%	55.8% versus 13.2%
STEP-4^48^ (n=803)	Age: 46 ± 12 years Weight: 107.2 ± 22.7 kg BMI: 38.4 ± 6.9 kg/m^2^	Placebo	-7.9% versus +6.9%* -17.4% versus -5.0% (entire trial)	88.7% versus 47.6% (entire trial)	79.0% versus 20.4%	63.7% versus 9.2%

*Data represent mean ± standard deviation.*

*Mean weight changes from week 20.

*BMI = body mass index; STEP = Semaglutide Treatment Effect in People with obesity; T2D = type 2 diabetes.*

**Table 3:** Summary of STEP programme (ongoing studies)^49–53^

Trial^Ref^	Clinical Trial Number	Title	Comparator	Completion date
STEP-5^49^ N=304	NCT03693430	Two-year effect and safety of semaglutide 2.4 mg once-weekly in subjects with overweight or obesity	Placebo	March 2021
STEP-6^50^ N=401	NCT03811574	Effect and safety of semaglutide once-weekly in East Asian subjects with overweight or obesity	Placebo	November 2020
STEP-7^51^ N=375	NCT04251156	Effect and safety of semaglutide 2.4 mg once-weekly on weight management in subjects with overweight or obesity (with or without T2D)	Placebo	October 2022
STEP-8^52^ N=338	NCT04074161	Effect and safety of subcutaneous semaglutide 2.4 mg once weekly compared to liraglutide 3.0 mg once daily on weight management in subjects with overweight or obesity	Liraglutide 3 mg, placebo	May 2021
SELECT^53^ N=17,500	NCT03574597	SELECT – Semaglutide effects on cardiovascular outcomes in people with overweight or obesity Duration: 59 months	Placebo	September 2023

*SELECT = Semaglutide Effects on Cardiovascular Outcomes in People With Overweight or Obesity; STEP = Semaglutide Treatment Effect in People with obesity; T2D = type 2 diabetes.*

The Semaglutide treatment effect in people with obesity (STEP) programme consists of eight clinical trials (STEP 1–8) investigating the effect of once-weekly injectable semaglutide 2.4 mg on weight loss, safety and tolerability in adults who are obese or overweight (*Tables 2* and *3*).^44–53^ All STEP trials include participants without T2D except STEP-2 and STEP-7. The STEP studies are randomized, double-blind, placebo-controlled trials except for STEP-2, in which comparators are semaglutide 1 mg and placebo, and STEP-8, in which comparators are liraglutide 3 mg and placebo. The primary endpoint for all trials is change in body weight from baseline to end of treatment. To date, four STEP trials (1–4) have been published, supporting the use of semaglutide 2.4 mg in weight management.^45–48^ Subsequently, in June 2021, the FDA approved Wegovy^®^ (semaglutide) injection (2.4 mg once weekly semaglutide) for chronic weight management in adults with obesity or overweight with at least one weight-related condition (such as hypertension, T2D or dyslipidaemia) for use in addition to a reduced calorie diet and increased physical activity.^19^

In the STEP-1 trial, 1,961 participants who were overweight or obesity but without T2D were enrolled to receive once-weekly subcutaneous semaglutide 2.4 mg or placebo plus lifestyle intervention for 68 weeks.^45^ At baseline, participants had a mean body weight of 105.3 ± 22.3 kg and BMI of 37.9 ± 6.6 kg/m^2^. At 68 weeks, the mean change in body weight from baseline was -14.9% in the semaglutide group compared with -2.4% in the placebo group, with the estimated treatment difference of -12.4% (95% confidence interval [CI], -13.4 to -11.5; p<0.001). In the semaglutide group, 86.4% (versus 31.5%), 69.1% (versus 12.0%) and 50.5% (versus 4.9%) achieved ≥5%, ≥10% and ≥15% body weight loss, respectively, compared with the placebo group. Treatment discontinuation rate was greater in the semaglutide group (4.5% versus 0.8%) due to gastrointestinal events, with nausea and diarrhoea being the most common adverse events.

The STEP-2 trial was a randomized, double-blind, double-dummy, placebo-controlled trial investigating semaglutide 2.4 mg once weekly versus semaglutide 1 mg once weekly versus placebo in 1,210 participants who were overweight or obese and had T2D.^46^ Primary endpoints were percentage change in body weight and achievement of weight reduction of at least 5%. At 68 weeks, the estimated change in mean body weight from baseline was -9.6%, -7.0% and -3.4% with semaglutide 2.4 mg, semaglutide 1 mg and placebo, respectively. The estimated treatment difference for semaglutide 2.4 mg versus placebo was -6.2% (95% CI, -7.3 to -5.2; p<0.0001). More participants in the semaglutide 2.4 mg group than the placebo group achieved weight reductions of at least 5% (68.8% versus 28.5%; odds ratio 4.88, 95% CI, 3.58 – 6.64; p<0.0001). Similarly, more participants achieved weight reduction of at least 10% (45.6% versus 28.7% versus 8.2%), 15% (25.8% versus 13.7% versus 3.2%) or 20% (13.1% versus 4.7% versus 1.6%) with semaglutide 2.4 mg compared with semaglutide 1 mg or placebo. Gastrointestinal adverse events were more frequent with semaglutide 2.4 mg and 1 mg than with placebo (63.5% versus 57.5% versus 34.3%, respectively).

The STEP-3 trial was designed to compare the effect of semaglutide versus placebo as an adjunct to intensive behavioural therapy on body weight in people who were overweight or obese but without T2D.^47^ Participants were randomized to subcutaneous semaglutide 2.4 mg (n=407) or placebo (n=204). Both groups received a low-calorie diet for the first 8 weeks and intensive behavioural therapy (30 counselling sessions) for the 68-week trial period. In line with the STEP-1 and STEP-2 trial findings, the semaglutide group observed greater weight loss: the estimated treatment difference between semaglutide and placebo was -10.3% (95% CI, -12.0 to -8.6; p<0.001). A higher proportion of participants in the semaglutide versus placebo group achieved weight losses of at least 5%, 10% or 15% (86.6% versus 47.6%, 75.3% versus 27.0% and 55.8% versus 13.2%, respectively; p<0.001). The number of gastrointestinal adverse events (82.8% versus 63.2%), as well as treatment discontinuation rate (3.4% versus 0%), were higher for participants treated with semaglutide compared with placebo.

The STEP-4 trial aimed to evaluate the effect of semaglutide against placebo on weight loss maintenance.^48^ In this study, participants received once-weekly semaglutide during the run-in phase. After 20 weeks, participants who reached the 2.4 mg maintenance dose (n=803) were randomized to continue 2.4 mg semaglutide for a further 48 weeks or switch to placebo, plus lifestyle intervention were conducted in both groups. The primary endpoint was percentage change in body weight from week 20 to week 68. Mean body weight change from week 20 to week 68 was -7.9% versus +6.9% in the continued semaglutide group versus placebo group, respectively, with the difference of -14.8% (95% CI, -16.0 to -13.5; p<0.001). Waist circumference and systolic blood pressure improved with continued semaglutide versus placebo. There were improvements in both the physical (0.8 versus -0.9, p=0.002) and mental (0.1 versus -3.4, p<0.001) component summary scores of the 36-Item Short Form Health Survey with continued semaglutide versus placebo. Although gastrointestinal adverse events were more common with semaglutide (49.1% versus 26.1% placebo), the treatment discontinuation rate was comparable (2.4% versus 2.2%, respectively).

The observed weight loss with semaglutide in the STEP studies was greater than that seen with liraglutide in the SCALE Obesity and Prediabetes study, or any other pharmacotherapy.^40^ In addition, semaglutide in the STEP studies maintained the weight loss at 68 weeks, supporting its use in the long-term management of obesity. Nevertheless, differences in study populations, trial duration and prevalence of prediabetes between the studies preclude direct comparison. We eagerly await the results of the STEP-8 study, which is a head-to-head trial of semaglutide 2.4 mg once weekly versus liraglutide 3 mg once daily.^52^

Available evidence from the STEP studies suggests that semaglutide may have the potential to bridge the gap between pharmacotherapy and bariatric surgery. The degree of weight loss observed in the STEP studies is comparable to that with some bariatric procedures.^54^ In the Swedish Obese Subjects study, maximal weight loss observed after 1–2 years of vertical banded gastroplasty and laparoscopic adjustable gastric banding was 25% and 20%, respectively.^54^ In the STEP-4 trial, 40% of participants who were on semaglutide for 68 weeks lost 20% or more of their initial body weight.^48^ Further comparisons with bariatric surgery would be useful to validate the relative impact of injectable semaglutide as an obesity treatment. Trials exploring the weight loss impact of oral semaglutide in non-diabetic populations would also be of value, given the effect of injectable semaglutide and the likelihood of greater patient acceptability and medication adherence with the oral formulation in real life. Of interest, evidence from ongoing SURPASS studies has demonstrated the impressive weight loss effect of tirzepatide, which produced a progressive and dose-dependent weight loss of 11–14% at 40–52 weeks.^37,38^ The SURMOUNT trials investigating the efficacy and safety of tirzepatide for weight management are ongoing.^55,56^

## Cost-effectiveness of GLP-1RAs in obesity

With the ever-increasing financial pressures faced by health systems worldwide, the cost-effectiveness of pharmacotherapy for obesity is important. In the UK, NICE generally accepts an incremental cost-effectiveness ratio of up to £20,000 per QALY gained. Recently, NICE supported the use of liraglutide for weight loss, though only in people with a BMI ≥35 kg/m^2^ with prediabetes and high cardiovascular risk and when prescribed by a tier 3 weight management service.^18^ This was following a confidential price reduction for purchases from such weight management services, which brought the cost per QALY to £11,293.^18^

Semaglutide is not currently recommended by NICE for the treatment of obesity without T2D in the UK, though an appraisal of the clinical and cost-effectiveness of this GLP-1RA recently started, and publication is expected within the next 12 months.^57^ One study based on the Research study comparing a new medicine semaglutide to liraglutide in people with type 2 diabetes (SUSTAIN 10) trial found that the use of injectable semaglutide 1 mg weekly rather than liraglutide 1.2 mg daily for people with T2D resulted in a lifetime cost saving of £140 per patient.^58^ This cost saving was driven by a reduction in diabetes-related complications associated with the greater reductions in HbA1c and body weight.^58^ However, for the treatment of obesity, semaglutide was found to be not as cost-effective as other obesity pharmacotherapy due to its relatively higher costs, despite its more significant clinical impact than other drug therapies for obesity.^59^ Of note, the abovementioned studies do not include evidence from recently published STEP studies. Further cost-effectiveness studies of semaglutide in people with obesity will be supported by the ongoing SELECT (Semaglutide effects on heart disease and stroke in patients with overweight or obesity; ClinicalTrials.gov identifier: NCT03574597) study investigating major adverse cardiovascular outcomes in people who were overweight or obese without T2D.^53^ The SELECT study aims to recruit 17,500 participants, and the estimated study duration for an individual participant is from 31 to 59 months. The primary outcome measure is time-to-first-occurrence of a composite endpoint consisting of cardiovascular death, non-fatal myocardial infarction or non-fatal stroke. It is estimated to be completed by September 2023. We await the outcome of the ongoing NICE appraisal with great interest.

## Conclusions

Over the past two decades, GLP-1RAs have shown great promise in the treatment of people with T2D. Benefits beyond improved glycaemic control, including weight loss, improved cardiovascular risk factors, markers of fatty liver and renal disease and, most importantly, cardiovascular morbidity and mortality, make them a particularly attractive therapeutic option. The impact of GLP-1RAs, particularly liraglutide and semaglutide, on weight loss should guide clinicians to choosing these drugs when considering pharmacotherapy for people with T2D and obesity. However, recent trial data indicate that even in people without T2D, there is high-quality evidence to support their use for weight loss. With the promising results from the STEP 1–4 trials, semaglutide has been added to the pharmacotherapy of weight management. Additionally, given the promise of the dual GIP/GLP-1 agonist tirzepatide for the treatment of obesity in people with T2D, further trials exploring cardiovascular outcomes with this drug would be welcome to support its use in routine clinical practice.

Ongoing cost-effectiveness analyses of these drugs in various populations and subpopulations are essential to validate the health economic impact of these drugs. The SELECT study may further justify the use of semaglutide in people with obesity to improve cardiovascular morbidity and mortality and thereby influence its incremental cost-effectiveness ratio. Clinicians should be aware of these findings and consider the use of these drugs when appropriate to improve health outcomes and reduce the prevalence of obesity-related disability, which is frequently observed in people with T2D.
